# Ceramide-1-Phosphate as a Potential Regulator of the Second Sodium Pump from Kidney Proximal Tubules by Triggering Distinct Protein Kinase Pathways in a Hierarchic Way

**DOI:** 10.3390/cimb44030066

**Published:** 2022-02-22

**Authors:** Lindsey M. P. Cabral, Fernando G. Almeida, Gloria M. R. S. Grelle, Adalberto Vieyra, Celso Caruso-Neves, Marcelo Einicker-Lamas

**Affiliations:** 1 Laboratório de Biomembranas, Instituto de Biofísica Carlos Chagas Filho, Universidade Federal do Rio de Janeiro, Ilha do Fundão, Rio de Janeiro 21941-902, Brazil; lindseycabral@yahoo.com.br (L.M.P.C.); fernandogalmeida@gmail.com (F.G.A.); gloriagrelle@biof.ufrj.br (G.M.R.S.G.); 2 Laboratório de Físico-Química Biológica Aída Hasson Voloch, Instituto de Biofísica Carlos Chagas Filho, Universidade Federal do Rio de Janeiro, Ilha do Fundão, Rio de Janeiro 21941-902, Brazil; avieyra@biof.ufrj.br; 3 Centro Nacional de Biologia Estrutural e Bioimagem/CENABIO, Universidade Federal do Rio de Janeiro, Ilha do Fundão, Rio de Janeiro 21941-902, Brazil; 4 Laboratório de Sinalização Celular, Instituto de Biofísica Carlos Chagas Filho, Universidade Federal do Rio de Janeiro, Ilha do Fundão, Rio de Janeiro 21941-902, Brazil; caruso@biof.ufrj.br

**Keywords:** Na^+^-ATPase, ceramide-1 phosphate, kidney, proximal tubules, basolateral membranes, sphingolipids

## Abstract

Kidney proximal tubules are a key segment in the reabsorption of solutes and water from the glomerular ultrafiltrate, an essential process for maintaining homeostasis in body fluid compartments. The abundant content of Na^+^ in the extracellular fluid determines its importance in the regulation of extracellular fluid volume, which is particularly important for different physiological processes including blood pressure control. Basolateral membranes of proximal tubule cells have the classic Na^+^ + K^+^-ATPase and the ouabain-insensitive, K^+^-insensitive, and furosemide-sensitive Na^+^-ATPase, which participate in the active Na^+^ reabsorption. Here, we show that nanomolar concentrations of ceramide-1 phosphate (C1P), a bioactive sphingolipid derived in biological membranes from different metabolic pathways, promotes a strong inhibitory effect on the Na^+^-ATPase activity (C1P_50_ ≈ 10 nM), leading to a 72% inhibition of the second sodium pump in the basolateral membranes. Ceramide-1-phosphate directly modulates protein kinase A and protein kinase C, which are known to be involved in the modulation of ion transporters including the renal Na^+^-ATPase. Conversely, we did not observe any effect on the Na^+^ + K^+^-ATPase even at a broad C1P concentration range. The significant effect of ceramide-1-phosphate revealed a new potent physiological and pathophysiological modulator for the Na^+^-ATPase, participating in the regulatory network involving glycero- and sphingolipids present in the basolateral membranes of kidney tubule cells.

## 1. Introduction

From the initial studies in the 90s, bioactive sphingolipids have emerged as an important class of cell signaling molecules, which are rapidly mobilized upon cell stimulation and are able to trigger different cell signaling cascades that culminate with specific cell responses [1,2].

Among the bioactive sphingolipid, there is increased interest in sphingosine (Sph), sphingosine-1-phosphate (S1P), ceramide (Cer), and ceramide-1-phosphate (C1P), which are those related to the so called “sphingolipid rheostate” involved in opposite effects such as cell survival or death, according to the appropriate switch [3,4].

The different bioactive sphingolipids involved in these signaling cascades can be mobilized either by de novo synthesis or by direct triggering the membrane phospholipid precursor, mainly sphingomyelin (SM) [5,6,7]. Different reports show that an initial step to cell signaling is the activation of sphingomyelinases (SMases), which cleaves SM, leading to the formation of Cer, which can either be direct involved in the assembly of structurally and functionally complex membrane microdomains called platforms [8,9,10], or as the substrate for a ceramide kinase (CerK), leading to the formation of C1P [11,12,13]. In a posterior step, C1P can be dephosphorylated to Cer, which is converted to Sph by the action of ceramidases [4,7,14,15]. This pathway starting in SM leads to the formation of two important mediators that are substrates for lipid kinases, resulting in phosphorylated derivatives with distinct abilities [1,16,17]. First, it was thought that the phosphorylation of the lipid mediators was related to the termination of the signal. Otherwise, the progress in the field allowed us to show that the phosphorylated derivatives, and here we will highlight C1P, are able to elicit distinct cell responses by direct interactions with hydrophobic domains including C1 and C2 that are present in different possible cellular partners [18,19,20,21,22], or, acting through the activation of still unknown membrane receptors, as already suggested [4,23].

In the last fifteen years, our group has been studying the ability of bioactive lipids to modulate some of the main ion transporters and other cell signaling pathways present in the basolateral membranes (BLM) from kidney proximal tubules [18,24,25,26,27,28,29]. Here, we extend our efforts to bring new insights into the role of C1P within the renal tissue, with C1P the bioactive lipid less studied in the sphingolipid rheostate scenario, thus illustrating the need to identify intracellular target molecules/pathways and intracellular mechanism(s) for C1P, and also uncover any crosstalk with other bioactive lipids [30]. The ability of Cer and C1P in triggering different protein kinases, and the increasing list of effectors shown to be modulated by these sphingolipids [2,12,31,32] allowed us to hypothesize that both bioactive sphingolipids could also be responsible for the regulation of Na^+^ transporters in the BLM. This could be particularly interesting not only due to the capacity of ceramides triggering effector proteins, as already demonstrated by our group [18,33], but also due to the importance of Cer for the assembly of membrane rafts and caveolae as well as large membrane microdomains, so called platforms, which are specific regions in plasma membranes where signaling molecules and effectors are co-localized [8,9,34,35].

Being the proximal tubule, the main nephron segment referred to Na^+^ reabsorption, and with C1P potentially available and/or recruited to this niche, here, we explore the action of C1P in different kinases that are known to modulate Na^+^ + K^+^-ATPase and the ouabain-insensitive, K^+^-insensitive, and furosemide-sensitive Na^+^-ATPase (referred from now on as Na^+^-ATPase), thus studying the regulation of these Na^+^ transporters in the BLM by C1P and also unrevealing the cell signaling cascades involved. From the results presented here, these described pathways involved in the regulatory network responsible for Na^+^ reabsorption may provide some means for the development of widespread new therapeutic strategies for different renal diseases and also more complex pathologies such as hypertension.

## 2. Materials and Methods

### 2.1. Material

General chemicals used were obtained from Sigma Chemical Co. (St. Louis, MO, USA) Percoll was obtained from Pharmacia (Uppsala, Sweden). Milli-Q system of resins (Millipore Corp., Bedford, MA, USA) applied to obtain deionized water used to prepare all solutions. ^32^P_i_ was obtained from the Instituto de Pesquisas Energéticas e Nucleares (IPEN, São Paulo, Brazil). [γ-^32^P]ATP was prepared as described previously [36]. Cer (from bovine brain), C1P, histone H8, the PKA α-catalytic subunit inhibitor 5–22 peptide (PKAi), and the PKC inhibitor calphostin C was bought from Sigma Chemical Co. (St. Louis, MO, USA). Reagents were of the highest purity available. The ECL^TM^ system and nitrocellulose membranes (Hybond) were obtained from GE Healthcare, (São Paulo, Brazil). Pig kidneys were purchased as already described [28], according to the respective Brazilian law.

### 2.2. Isolation of Basolateral Membranes (BLM)

Kidneys were rapidly transported in a chilled solution (250 mM sucrose, 10 mM Hepes–Tris—pH 7.6—2 mM EDTA, 1 mM PMSF, and 0.15 mg/mL of soybean trypsin inhibitor). The *cortex corticis* was carefully removed and enriched basolateral membrane (BLM) fractions derived from kidney proximal tubules were prepared as previously described [37]. Controls for contamination with other membranes were carried out as previously described [38]. Na^+^ + K^+^-ATPase activity was enriched up to six-fold (297.2 ± 1.2 nmol/mg per min) over the initial cortex homogenate. The BLM aliquots were stored in 250 mM sucrose in liquid N_2_.

### 2.3. Protein Determination

Protein determination assays were performed by the Folin phenol method [39] with the addition of 5% SDS in the samples using bovine serum albumin as the standard.

### 2.4. Determination of Na^+^ + K^+^-ATPase and Na^+^-ATPase Activities

The assays were carried out in a reaction medium (1 mL final volume) containing 50 mM Bis-Tris propane buffer (pH 7.4), 5 mM MgCl_2_, 10 mM NaN_3_, 20 μM free Ca^2+^ (0.2 mM EGTA, 238.3 μM CaCl_2_) where the BLM fractions (0.2 mg/mL) were preincubated for 10 min at 37 °C with the C1P concentrations indicated in the figures, followed by 10 min sonication (240 W, 25 kHz, 24–25 °C; Unique Sonifier Cleaner). Then, the samples were supplemented with 120 mM KCl and 5 mM ATP. When indicated, 10 nM PKAi (5–22 inhibitor peptide of PKA) or 10 nM calphostin C (PKC inhibitor) were added to the preincubation medium. After 20 min, 1.5 mL activated charcoal in 0.1 N HCl was added to each tube and mixed in a vortex to stop the reaction and followed by centrifugation (2000 rpm). The 0.5 mL aliquots of the supernatants were transferred to new glass tubes, and the amount of Pi released was determined using a colorimetric method [40]. Na^+^ + K^+^-ATPase activity was calculated as the difference between the activities measured without and with 1 mM ouabain [35,38]. Na^+^-ATPase activity was assayed using the same reaction medium for Na^+^ + K^+^-ATPase activity determination with [γ-32 P]ATP as the substrate for the ATPase (108 cpm/reaction tube). The Na^+^-ATPase activity was calculated as already described [38,41]. Briefly, the reaction was stopped after 20 min by adding the activated charcoal to each tube, followed by centrifugation (2000 rpm). The 0.5 mL aliquots of the supernatants were dispensed to filter paper strips and the released [32 P]Pi was determined by liquid scintillation counting. Na^+^-ATPase activity was determined as the difference between the activities measured without and with 2 mM furosemide, in the presence of 1 mM ouabain.

The free Ca^2+^ concentration for these determinations was calculated using a computer program that took into account the different species involved in the equilibrium between EGTA, Ca^2+^, and the different ATP forms, Mg^2+^, H^+^, K^+^ [42]. 

### 2.5. Determination of Protein Kinase A (PKA) Activity

Histone H8, a general substrate for protein kinases, was used in the BLM PKA activity assay, as already described [18]. The PKA activity was calculated as the difference between the total radioactivity associated with the histones in the presence or absence of PKAi (10 nM). A total of 100 nM cAMP was used as a positive control to confirm the presence of PKA in the BLM fractions.

### 2.6. Determination of Protein Kinase C (PKC) Activity

We used the same method described for the determination of PKA activity [18], and PKC activity was measured as the difference between the histone phosphorylation in the presence or absence of 10 nM calphostin C (PKC inhibitor). Phorbol-12-myristate-13-acetate (PMA, 1 pM), a phorbol ester that is a DAG analogue, was used to confirm the presence of functional PKC in the BLM fractions [35].

### 2.7. Statistical Analysis 

Statistical analysis was carried out using the one-way ANOVA test and Newman–Keuls post-test. Statistical significance was set at *p* < 0.05. Data were analyzed using the GraphPad Prism 5.0 program.

## 3. Results

**Figure 1:**
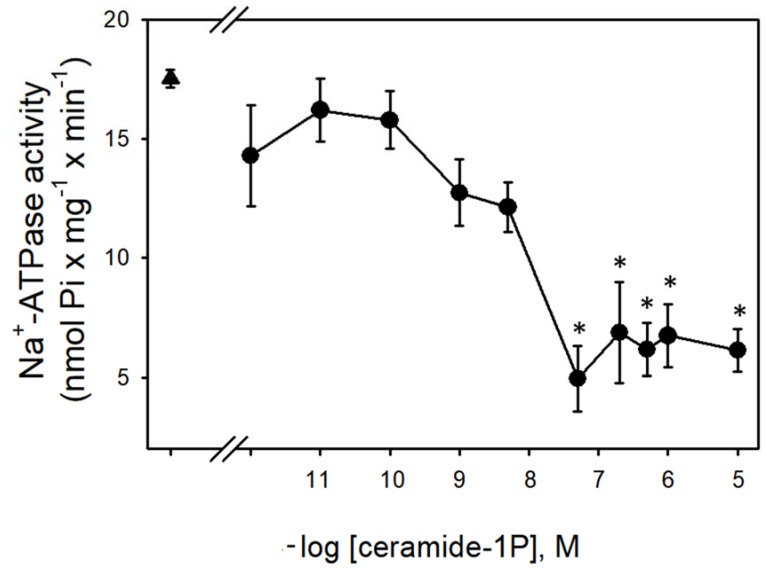
Ceramide-1-phosphate inhibits BLM Na^+^-ATPase activity. Na^+^-ATPase activity assays were performed in the presence of crescent C1P concentrations shown on the abscissa (see details in Material and Methods). Data are means ± SE of at least six experiments conducted in triplicate with different kidney BLM preparations. Triangle: control without C1P. * Significantly different from the control (*p* < 0.05).

**Figure 2:**
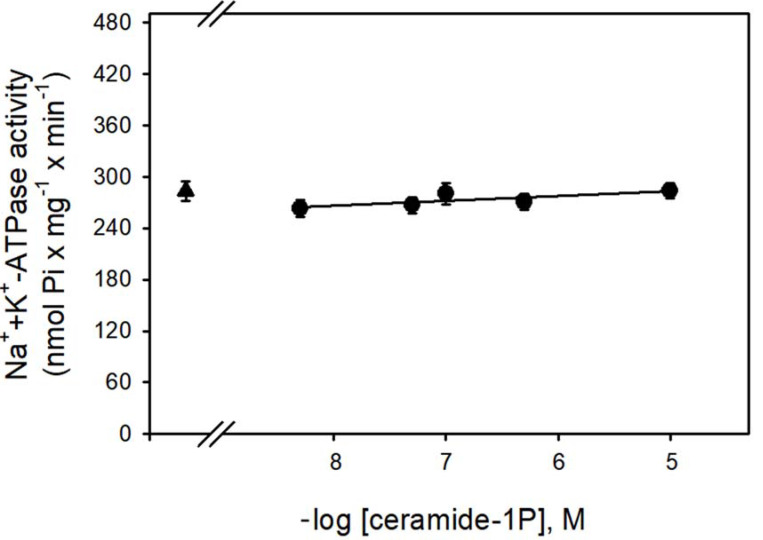
The BLM Na^+^/K^+^-ATPase was not modulated by C1P. The Na^+^/K^+^-ATPase activity was determined as described in the Material and Methods, in the presence of crescent C1P concentrations shown on the abscissa. Data are means ± SE of at least six experiments performed in triplicate with different kidney membrane preparations. Triangle: control without C1P.

Figure 1 shows that the Na^+^-ATPase activity from BLM was significantly inhibited by increasing concentrations (in the nanomolar range) of C1P, with the effect of a physiological effectiveness as judged by the relative low concentration (≈10 nM) that almost abolished the Na^+^-ATPase activity. Due to our interest in the regulation of Na^+^ transport by C1P, we also tested the ability of this bioactive sphingolipid in the modulation of the Na^+^ + K^+^-ATPase. As shown in Figure 2, C1P was ineffective in modulating Na^+^ + K^+^-ATPase activity, even at those higher concentrations tested. 

The significant modulatory effect of C1P on the Na^+^-ATPase activity could be ascribed to (i) structural changes in the BLM similar to the observed upon Smase activation and Cer release [31,43] or (ii) to a direct activation of protein kinases that are known as targets for ceramides in different cells [12,18,44]. In a previous paper, we showed that Cer was able to modulate PKC and PKA in the BLM [18,33]. Therefore, since PKC and PKA were notably involved in the modulation of Na^+^-ATPase [45,46,47,48], the following experiments were conducted to investigate whether the inhibitory effect of C1P on active Na^+^ transporters in kidney proximal tubules involved PKC or PKA, two of the major kinases already shown by our group within the renal BLM [18,33].

**Figure 3:**
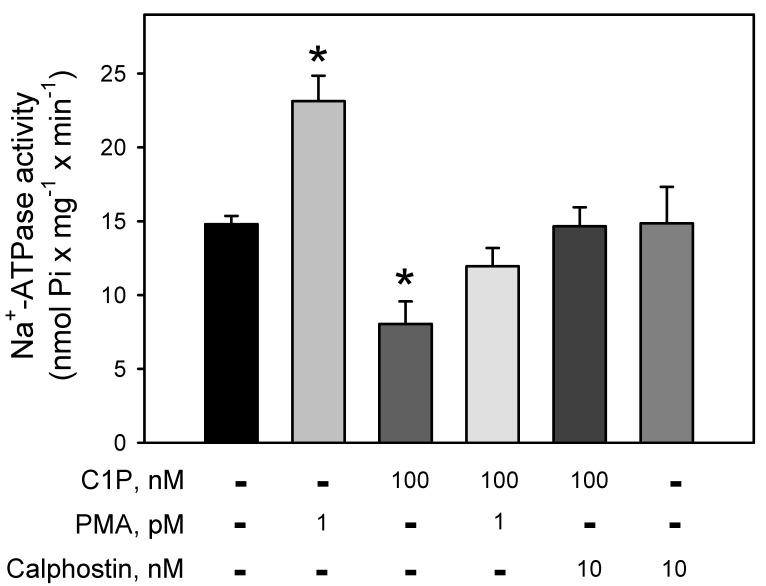
C1P inhibition of the Na^+^-ATPase activity is completely abolished by pre-incubation of the BLM with calphostin, a specific PKC inhibitor. Na^+^-ATPase activity was assayed in the presence of 100 nM C1P, phorbol myristate acetate (PMA) 1 pM, and calphostin C 10 nM alone or in the different combinations shown on the abscissa. Data are expressed as means ± SE of at least six experiments conducted in triplicate using BLM fractions from different kidney preparations. * Statistical difference related to the control (*p* < 0.01).

Figure 3 clearly demonstrates the involvement of a BLM-PKC in the modulation of the Na^+^-ATPase. The pump is strongly stimulated (≈64%) by PMA, a phorbol ester that mimics DAG within the reaction medium, leading to the PKC activation. Conversely, C1P induced a significant inhibition (≈43%) on the Na^+^-ATPase activity, which is fully prevented when the BLM fractions were pre-incubated with the well-known PKC-inhibitor, calphostin-C. Simultaneous addition of PMA and C1P had no effect on the ATPase activity, suggesting a mutual counteraction on the pump.

**Figure 4:**
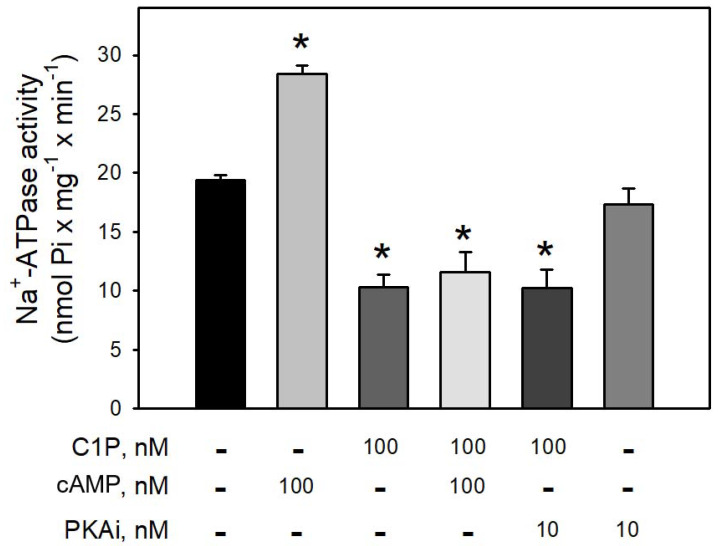
The PKA inhibitor (5–22 peptide) was not able to prevent the inhibition of Na^+^-ATPase activity by C1P. Na^+^-ATPase activity was measured as described in the Material and Methods. The assays were carried out in the presence of 100 nM C1P, 100 nM cAMP, and 10 nM PKA_i_ (5–22 peptide) individually tested or in the combinations shown on the abscissa. Data are expressed as means ± SE of at least six experiments conducted in triplicate with BLM from different kidney preparations. * Statistically different when compared to the control (*p* < 0.01).

Figure 4 clearly shows that preincubation of BLM with cAMP led to a robust increase (≈65%) in the Na^+^-ATPase activity, suggesting that BLM-associated PKA are ready to be activated and trigger the second sodium pump. On the other hand, preincubation of the BLM fractions with 100 nM C1P led to a significant inhibition (≈40%) of the pump, which was not reversed in the presence of cAMP, or prevented by preincubating the BLM fractions with 10 nM PKAi. As expected, the pre-incubation of the BLM with the PKAi had no effect on the Na^+^-ATPase activity.

**Figure 5:**
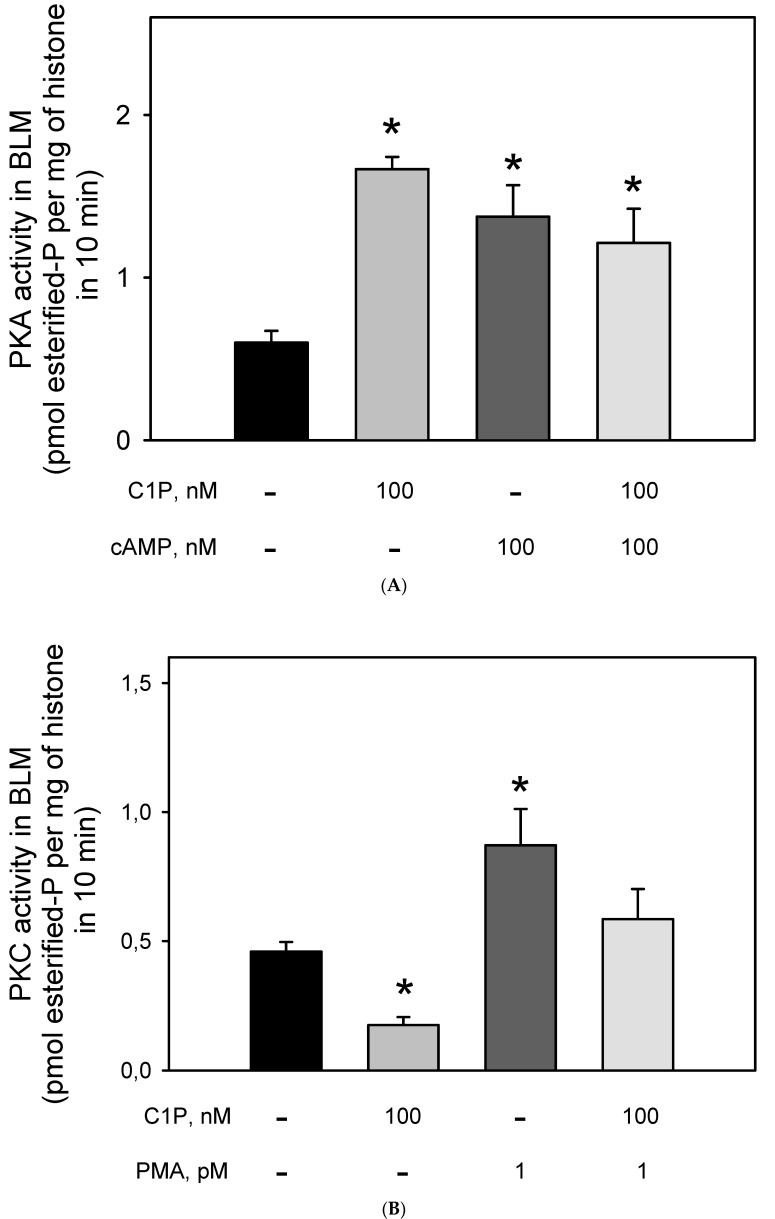
Ceramide-1 phosphate differentially modulates PKC and PKA in the BLM. (**A**) PKA_i_-inhibited kinase activity was assayed as described in the Material and Methods, either in the absence or presence of C1P and cAMP added alone, or in the combinations shown on the abscissa. PKA activity was determined as the difference between total histone H8 phosphorylation and that measured in the presence of 10 nM PKA_i_ (5–22 peptide). (**B**) Calphostin C-inhibited kinase activity was assayed as described in the Material and Methods either in the absence or presence of C1P and PMA added alone or in the combinations shown on the abscissa. PKC activity was determined as the difference between total histone H8 phosphorylation and that measured in the presence of Calphostin C. Data are the means ± SE of at least six experiments performed in triplicate with different kidney membrane preparations. * Statistical difference with respect to the control without additions (*p* < 0.01).

The effect of Cer on the BLM-PKA and -PKC activity was previously demonstrated [18,33], but to our knowledge, there were no data on the action of C1P on the BLM-associated kinases. Thus, we decided to evaluate the PKA and PKC activities after stimulation of the BLM with C1P. Figure 5A shows that the incubation of the BLM fractions with 100 nM of C1P led to a two-fold increase in the BLM-associated PKA activity, which was the same as that obtained when the BLM fractions were incubated with 100 nM cAMP. Simultaneous addition of C1P and cAMP did not lead to an addictive effect, thus suggesting that C1P and cAMP activate the same BLM-associated PKA pool, or that if there are different PKA pools, they are activated to the same extent. On the other hand, Figure 5B shows that C1P significantly inhibited (by ≈43%) the BLM-PKC activity, with the effect being completely counteracted when 10^−12^ M PMA was simultaneously added to the assay. The results presented here clearly show that 100 nM cAMP and 1 pM PMA stimulated both the Na^+^-ATPase and respectively PKA and PKC to the same extent, while the regulatory network elicited by C1P could not be directly correlated. This led us to reinforce the hypothesis raised in a previous work [33] considering that there must exist a hierarchy between PKA and PKC activities triggering the Na^+^-ATPase within the BLM.

## 4. Discussion

**Figure 6:**
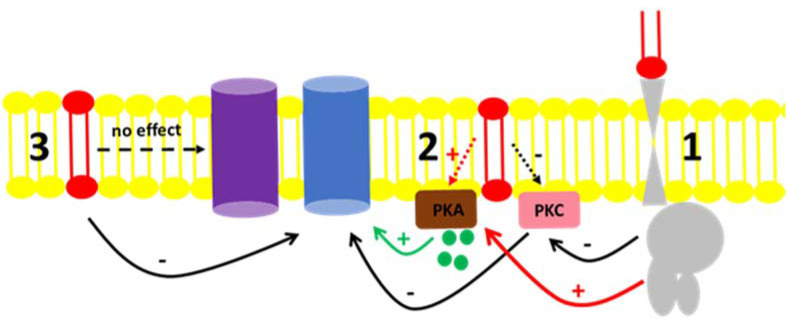
Proposed mechanism for the C1P action on the Na^+^-ATPase activity from BLM. (**1**) C1P (red lipid) inhibits PKC through a yet unknown G-protein coupled receptor, resulting in the inhibition of the Na^+^-ATPase (blue protein). The same route could activate BLM PKA (red arrow) with no effect on the pump. (**2**) C1P could directly bind to effectors present in the BLM, such as the PKC, leading to the inhibitory action on the Na^+^-ATPase activity described. Our results showed that although PKA is present in the BLM and ready to modulate the pump by cAMP (green circles and green arrow), its activation by C1P had no effect in the pump (red dashed arrow). (**3**) C1P directly binds to the Na^+^-ATPase or is enriched in the lipidic membrane microenvironment surrounding the Na^+^-ATPase, thus modulating it. From the results presented here, we were not able to detect any modulatory action of C1P on the Na^+^/K^+^-ATPase (purple protein) present in the BLM fractions.

Fluid transport across the tubular epithelium is one of the key events directly correlated to the majority of the distinct renal functions [49]. Among the different solutes handled in the kidney, Na^+^ is especially important. Due to its abundance in the extracellular fluid, it is imperative to understand the mechanisms by which Na^+^ is reabsorbed or excreted in urine because alterations in the interstitial and intravascular Na^+^ concentrations lead to changes in volume and might cause disturbances in blood pressure [50]. The regulation of Na^+^ levels in body liquid compartments are principally driven by primary active transporters with the classical Na^+^ + K^+^-ATPase [51] being involved in mass transport, while the “second” Na^+^ pump or Na^+^-ATPase is involved in the fine-tuned regulation of Na^+^ reabsorption [52,53]. There are many reports in the literature concerning the regulation of Na^+^ + K^+^-ATPase by hormones and autacoids [50,54] as well as the role of regulatory phosphorylation [50,55]. In contrast, fewer groups quote the regulation of Na^+^-ATPase as there is still some controversy concerning its identity, even though there were reports showing a furosemide-sensitive Na^+^-ATPase activity from mammals [41,56,57] to protozoa [58,59] as well as the cloning of the pump from different organisms [60,61,62,63]. From the results presented here, our group showed that not only are hormones and autacoids able to trigger cell signaling pathways that modulate the Na^+^-ATPase, but the effect of C1P exogenously added to the BLM fractions points to either a possible interaction of the bioactive lipid with the hydrophobic domains of regulators, or the pump itself; or, there must be a kind of C1P receptor in the BLM, not identified yet [20,22,23]. Figure 6 shows a proposed scheme to summarize these possible C1P ways of action. The ability of ceramides to trigger kinases and elicit different physiological response was reported in liver cells [24]. The authors not only showed the ability of ceramide-dependent modulation of the Na^+^/K^+^-ATPase and kinase regulation, but they also reported biphasic responses considering time-dependent stimulation of those cell lines, which illustrates that there must be a complex crosstalk between ceramide-dependent signaling and other signaling systems present in the cell membranes.

In the present work, we show that C1P inhibited the Na^+^-ATPase activity (C1P_50_ = 10 nM) (Figure 1), while it had no effect on the Na^+^+K^+^-ATPase resident in the same membranes (Figure 2). Moreover, the results presented here in combination with previous results from our group clearly show that Cer [33] and C1P modulated the BLM associated PKA and PKC, which appeared to mediate the effects elicited by ceramides on Na^+^-ATPase. When the BLM fractions were treated with 100 nM C1P, inhibition of the pump could not be attributed to PKA (Figure 4) since the pre-incubation with the PKAi had no effect on the C1P inhibitory action. On the other hand, the significative inhibition of the Na^+^-ATPase activity upon C1P treatment is fully prevented by calphostin C (Figure 3). Previous reports have shown that PKA stimulates the renal Na^+^-ATPase activity in a pathway that links G_s_-coupled receptors with the pump activity [41]. The signaling systems aborded here in our manuscript—PKA and PKC—have been well studied, so there is plenty information in the literature showing the concentrations for positive controls as used here (cAMP and phorbol myristate acid PMA, respectively). As our focus was not a dose-response study either with classic activators or inhibitors for the different signaling systems, we only used the concentrations of activators or inhibitors already effectively used in other studies from our or other groups. 

The detection of a hierarchy in the phosphorylation of the Na^+^-ATPase was suggested when we stimulated the BLM with Cer [33] and we observed that the blockage of the BLM-PKCζ blunted the inhibitory action of Cer on the pump and allowed cAMP-activated PKA to stimulate it. In spite of an increasing number of reports showing different roles for Cer activated kinases [44,64,65,66], this is the first report of a physiological action of C1P on BLM-resident kinases, which certainly plays a role in the regulation of Na^+^ handling in the kidney. We cannot definitively affirm that the Na^+^-ATPase is directly phosphorylated by the studied kinases, as there are no available experimental tools for this, mainly because the complete structure of the second sodium pump has not been identified yet. However, it is well known that the Na^+^-ATPase is a P-type ATPase, and this fact allows us to postulate that it can be directly modulated by phosphorylation in putative regulatory sites [46,50,55]. We cannot rule out that the activation of the respective kinases would lead to the phosphorylation of another protein in this regulatory network, which would be responsible for ATPase inhibition.

Among the different bioactive sphingolipid, C1P is less documented in the different physiological systems. On the other hand, sphingosine-1-phosphate, which may be the most important bioactive lipid, is very well studied in kidney physiology and pathophysiology [2,6,7,67]. As S1P and C1P members are a type of family of signaling molecules with common precursors and sharing mutual interconversion steps, it is possible to imagine that C1P would also be present along the entire nephron. The work by Sugiura et al., 2002 [11], a hallmark in the field, showed that CerK is highly expressed in kidneys. In spite of robust data in the literature showing the role of C1P in podocytes and glomerular disease [68,69], there has not been a work individually showing CerK expression and C1P action in any of the nephron segments. Otherwise, C1P is likely to be present in all the renal cells either by the presence of sphingomyelin, which would allow the salvage route for C1P synthesis, or by the fact that the C1P can be provided to the renal tissue by the blood stream. These are very plausible ways to think that this signaling lipid would be playing different roles in the other nephron segments, although we did not explore it here.

It was substantially demonstrated that Cer levels were significantly elevated during renal injury due to an already reported increase in Ca^2+^-stimulated sphingomyelinase activity after cellular Ca^2+^ homeostasis is disrupted [2,4,24,43,70]. This observation allows us to postulate that the kinase-mediated modulation of the BLM Na^+^-ATPase activity by Cer and C1P could be especially relevant in the injured renal tissue, what could open a new role for ceramides in the establishment and progression of nephropathies. In the presence of C1P, the BLM kinases studied were differentially modulated (Figure 5). While C1P inhibited the PKC, leading to a significant inhibition on the Na^+^-ATPase activity, C1P-dependent activation of PKA had no effect on the Na^+^-ATPase activity. This allows us to postulate that there is a hierarchy involving different kinases, which results in a complex switch in/off to the Na^+^-ATPase. This switch may involve different isoforms of PKC and PKA, or, there must be crosstalk with the potential conversion of C1P to Cer and other bioactive lipids triggering other signaling pathways harbored in the BLM, which has already been described in liver cells [24]. 

The balance between Cer and C1P could be a new promising target to the development of new classes of drugs with more efficient action to try to stop the progress of renal failure, or at least, raise the life quality for renal patients. 

## 5. Conclusions

From the results presented here, a signaling cascade starting with Cer production and further phosphorylation to C1P is an efficient pathway for inhibiting Na^+^-ATPase in renal proximal tubules cells through BLM associated PKA and PKC as effectors [17]. These observations reveal the importance of protein kinases in the fine control of Na^+^ fluxes in a nephron segment where more than two-thirds of the glomerular ultrafiltrate is reabsorbed, the proximal tubule. To the best of our knowledge, the present work shows for the first time that C1P can modulate the BLM-Na^+^-ATPase from kidney proximal tubule cells by triggering membrane-associated kinases (PKA and PKC). Thus, the above results add evidence to the view that Cer and C1P participates in the regulatory network of bioactive sphingolipids and glycerolipids resident in this nephron segment, which should be true for the overall nephron. This hypothesis can be supported by the view that Cer is a lipid virtually present in all the nephron segments as well as that the Cer availability would allow for the formation of C1P.

## Data Availability

Data supporting the reported results can be found in the original files at the Biomembranes Laboratory.
